# The heterogeneity and change in the urban structure of metropolitan areas in the United States, 1990–2010

**DOI:** 10.1038/s41597-019-0329-6

**Published:** 2019-12-16

**Authors:** Stefan Leyk, Deborah Balk, Bryan Jones, Mark R. Montgomery, Hasim Engin

**Affiliations:** 1 Department of Geography, University of Colorado, Boulder, USA; 2 CUNY Institute for Demographic Research and Baruch College, Marxe School of International and Public Affairs, City University of New York, New York, USA; 3 Stony Brook University and Population Council, New York, USA; 4 CUNY Institute for Demographic Research, New York, USA

**Keywords:** Geography, Environmental social sciences

## Abstract

While the population of the United States has been predominantly urban for nearly 100 years, periodic transformations of the concepts and measures that define urban places and population have taken place, complicating over-time comparisons. We compare and combine data series of officially-designated urban areas, 1990–2010, at the census block-level within Metropolitan Statistical Areas (MSAs) with a satellite-derived consistent series on built-up area from the Global Human Settlement Layer to create urban classes that characterize urban structure and provide estimates of land and population. We find considerable heterogeneity in urban form across MSAs, even among those of similar population size, indicating the inherent difficulties in urban definitions. Over time, we observe slightly declining population densities and increasing land and population in areas captured only by census definitions or low built-up densities, constrained by the geography of place. Nevertheless, deriving urban proxies from satellite-derived built-up areas is promising for future efforts to create spatio-temporally consistent measures for urban land to guide urban demographic change analysis.

## Introduction

In 2014, residents of urban areas were estimated to account for 54% of the world’s total population, a figure projected to grow to 66% by mid-century^1^. Hidden in such aggregate measures is the great variation across countries not only in the national definitions of urban, but also in the pace and nature of change in such definitions over time^2–4^. Definitional changes are in part the result of an evolution in the conceptualization of urban-ness, which has been accompanied by a growing capacity of statistical authorities to distinguish meaningfully between urban land cover and urban population. In many countries, including in the United States, contemporary definitions of urban-ness consider residents who participate in urban social and economic life either directly in their places of residence or indirectly through non-residential connections such as commuting.

The urban structure and population distribution of urban areas has profound implications for public health and environmental issues, public service provision, and many other aspects of urban sustainability. In the academic literature on such urban issues in the United States, a commonly-adopted reference unit is the Metropolitan Statistical Area (MSA), which is a collection of contiguous counties selected in part to capture such non-residential connections^5–11^. Researchers have not always heeded the repeated criticism that MSAs include large rural areas as well as urban land^12–14^. In this paper, we investigate the internal composition of MSAs and document changes in these important units over the three most recent population censuses: 1990, 2000, and 2010. By accounting for the internal heterogeneity of MSAs, we strive to better understand the complexities of metro areas and their evolution.

Urban census definitions in the United States now include characteristics of land use^3,15,16^. Increasingly, satellite-derived land-use data are being used as inputs to define urban locations; in one effort led by the European Commission, such satellite and census data are combined to generate a globally consistent characterization of the rural-urban continuum^17–19^. That effort, as does this study, uses data from the Global Human Settlement Layer (GHSL)^20,21^, a public-domain, spatially-detailed (30 m resolution) time series (1975, 1990, 2000, and 2014) of remote sensing derived measures of “built-up” land. With the aid of these fine-grained data, we examine US census measures of urban people and urban land within and across MSAs. Our aim is to assess whether GHSL-based measures of built-up land cover are in agreement with census-defined urban population estimates, and to characterize the areas of disagreement at fine granularity and at different points in time. We believe that such disagreements suggest new avenues for focused research.

While it is well known that built-up land data, such as the GHSL, cannot substitute for richly detailed census data, they can provide helpful additional information about the complexity and heterogeneity of urban areas^17,22,23^, adding value especially in countries and areas that are data-poor or whose settlement patterns do not fit neatly into conventional, binary, urban-rural classifications. Moreover, the combined distributions of urban land and population have the potential to benefit urban planning efforts^24^ and inform population-land use projections^25^, a much-needed input for climate forecasts among many other uses. Combinations of GHSL with gridded population data^26^ also form the basis for a new globally applicable statistical approach to modeling the urban continuum^27^. We expect that the findings of this study will provide a conceptual and technical foundation for comparing and integrating census-based data with remotely-sensed measures of urban land.

Accordingly, the form of data integration described herein allows us to ask three pointed research questions: First, within broader metro aggregates, can we construct consistent urban classes from population and building density measures that describe an urban continuum, despite changes in census unit boundaries over time? Second, what do these patterns tell us about the distribution of urban land and population within and across metropolitan areas of the US and how much heterogeneity is observed between MSAs? Third, what temporal patterns of change are revealed by the new measures?

**Fig. 1:**
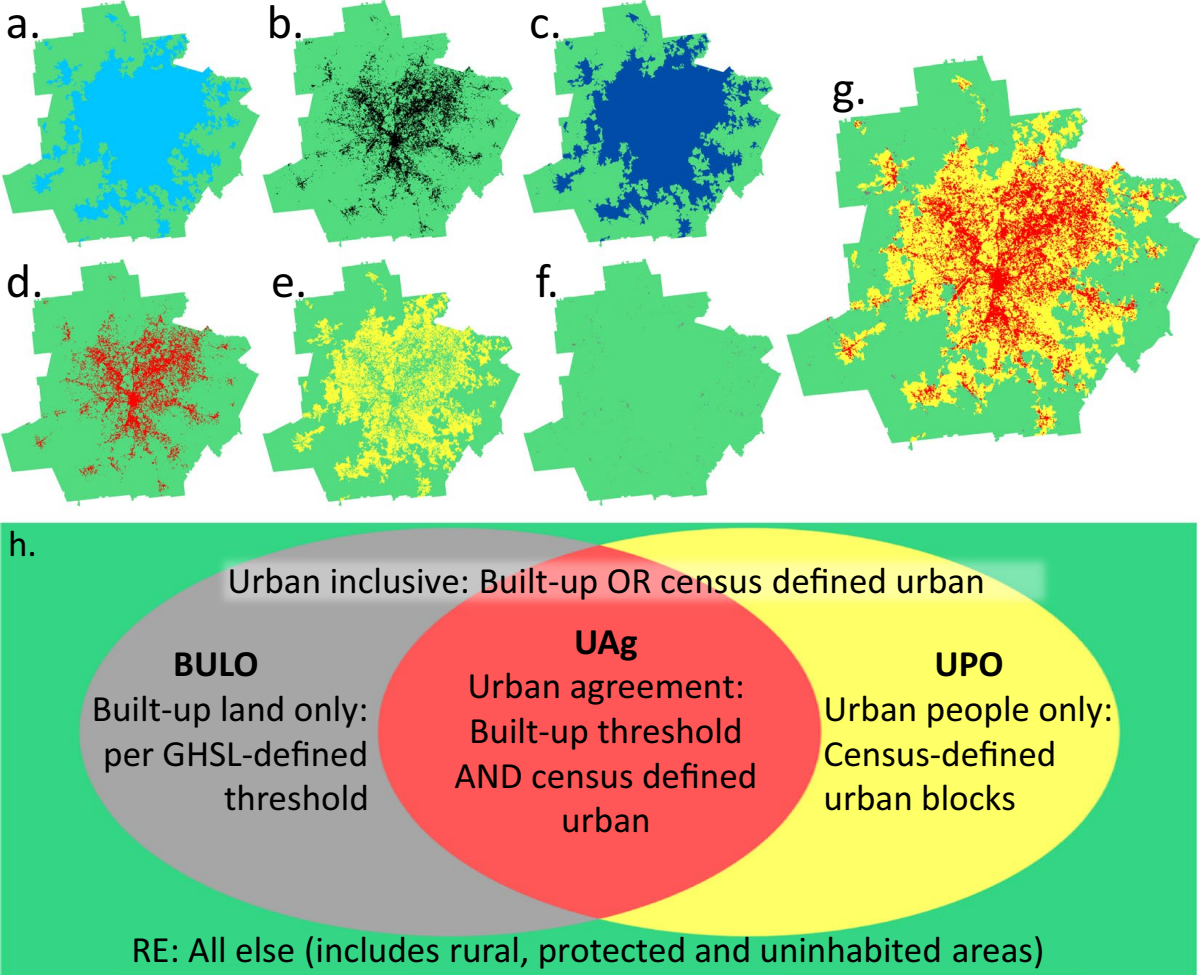
Layers for constructing urban classes shown for the Atlanta MSA, and in a conceptual overview. The layers shown are: (**a**) urban blocks, (**b**) all urban land (GHSL > 50% threshold), (**c**) urban inclusive area (*UI*), (**d**) urban agreement (*UAg*), (**e**) urban people only (*UPO*), (**f**) built-up land only (*BULO*), and (**g**) the entire urban hierarchy; green background indicates rural extents (*RE*); (**h**) conceptual overview of the classification schema resulting from combinations of the spatial layers *UAg, BULO, UPO* and *RE*.

We create three urban classes as depicted in Fig. 1: (1) Areas of Urban Agreement (*UAg*) meet a GHSL built-up threshold and are also officially defined as urban by US census criteria. Conceptually, we expect *UAg* to capture the urban core and closely surrounding areas. (2) Areas of Urban People Only (*UPO*) are those that do not meet the built-up threshold but are classified as urban by the census. We expect these to capture peri-urban and suburban locations, and identify what some might term ‘sprawl’. (3) Built-up land only (*BULO*) are areas that meet the GHSL built threshold, but are not classified as urban by the census. We expect these areas to be mostly in transition from census-defined rural to urban (at the fringes), or to represent isolated areas of (possibly new) economic activity.

The empirical work of this analysis sheds light on these conceptualizations: We evaluate the variability in population and area estimates of these urban classes for three points in time but within stable geometries of a combination of 2000 Metropolitan Statistical Areas (MSAs) and Consolidated Metropolitan Statistical Areas (CMSAs) (hereto with, MSA). The latter simplify the geography of larger metropolitan areas with populations greater than 1 million such that they can be treated as single units. Next, because metro- or city-size is often used as a marker for understanding differences in urban form, structure and sustainability^6,28^, we classify MSAs by population size and examine how the observed urban class variation aligns along size gradients. We then explore class distributions to expose heterogeneities across MSAs, as exemplified by the 10 largest MSAs and 10 smaller companion MSAs chosen for comparison.

## Results

### Different urban classes reveal distinct patterns and changes in land and population across MSAs

Table 1 shows population, land area and population density in each urban class and rural extents for 1990–2010 for all MSAs (fixed at 2000 Census boundaries) at the 50% GHSL threshold. The ***total population*** living in MSAs ranges from 197 million in 1990 (80% of the national total) to nearly 250 million (82% of the national total) in 2010. The majority (but not all) of the population within MSAs is defined as urban by the Census Bureau (composed of *UAG* and *UPO* class estimates: 85% in 1990, 89% in 2010). Using a GHSL built-up threshold of 50%, we find that of all people living in MSAs, from 71.5–74.1% live in urban built-up environments (*UAg*) depending on census year; some 27.5–25.4% live in officially urban but not built-up (*UPO*) areas; fewer than 1 percent inhabit *BULO* areas; and from 14.1–10.3% of MSA inhabitants live on rural land (Table 1).

**Table 1:** Population, land area and population density by urban classification for all MSAs, year-2000 MSA boundaries, 50% GHSL built-up threshold.

Fixed Geography		1990		2000		2010	
50% GHSL Threshold		Count	%	Count	%	Count	%
Population (000 s)	*Total MSAs*	*197*,*372*,*191*		*224*,*637*,*376*		*249*,*794*,*023*	
	Urban Inclusive (*UI*)	169,454,068	85.9%	199,462,646	88.8%	224,116,140	89.7%
	Urban Agreement	121,076,150	71.5%	147,263,592	73.8%	166,070,248	74.1%
	Urban People Only	46,671,239	27.5%	51,010,038	25.6%	57,026,153	25.4%
	Built-up Land Only	1,706,680	1.0%	1,189,017	0.6%	1,019,739	0.5%
	Rural Extents (*RE*)	27,893,410	14.1%	25,127,387	12.6%	25,677,624	10.3%
Area (km^2^)	*Total MSAs*	*1*,*857*,*315*		*1*,*855*,*901*		*1*,*856*,*519*	
	Urban Inclusive (*UI*)	193,811	10.4%	212,114	11.4%	242,656	13.1%
	Urban Agreement	66,261	34.2%	83,795	39.5%	102,075	42.1%
	Urban People Only	120,922	62.4%	117,285	55.3%	133,251	54.9%
	Built-up Land Only	6,628	3.4%	11,033	5.2%	7,330	3.0%
	Rural Extents (*RE*)	1,663,504	89.6%	1,642,471	88.5%	1,613,864	86.9%
Population Density (Persons/km²)	*Total MSAs*	106.3		121.0		134.5	
	Urban Inclusive (*UI*)	874		940.4		923.6	
	Urban Agreement	1,827		1,757.4		1,626.9	
	Urban People Only	386		434.9		428.0	
	Built-up land Only	257		107.8		139.1	
	Rural Extents (*RE*)	16.8		15.3		15.9	

Within MSAs, ***land area*** classified as *UAg* comprises 34% of the total urban area in 1990, rising to 42% by 2010. In contrast, within MSAs, areas defined as *UPO* comprise more than 62% of urban land in 1990, declining to 55% by 2010. Of all MSA land, however, 89.6–86.9% is in the rural category between 1990 and 2010, respectively.

***Population density*** varies significantly across classes, with the highest values seen for *UAg* and the lowest for *RE* (Table 1). Comparing 1990 to 2010, a slight downward trend in population density is evident within the *UAg* class, whereas a slight upward trend can be seen in the *UPO* class. As the table shows, the population of *UPO* areas has been growing, and as a result, *UPO areas* have also become more built-up, until they meet the criteria for *UAg*. The entry of formerly lower-density areas into the *UAg* class results in downward trends in *UAg* density over time. The trend in *BULO* is one of dramatic decline in density over time as these areas become increasingly defined as urban by the Census Bureau, a function of definitional changes in the census definition of urban (which is inclusive of remote sensing data in recent years). These results are based on a GHSL built-up threshold of 50%; had we adopted a more inclusive built-up threshold such as 25%, or even 1%, that would have resulted in a much larger fraction of the MSA population in areas of *UAg* by design, as well as greater shares of land area in both *UAg* and *BULO*.

### MSA population size matters, especially when decomposed by urban classes

**Fig. 2:**
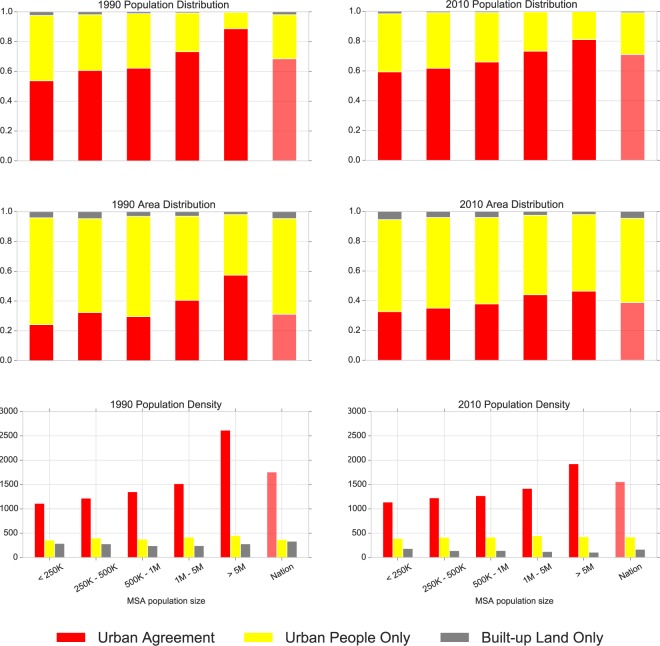
Population, land area proportions and population density by urban class and by MSA population size. Population (number of persons per class), land area proportions (total area per class in km^2^) and population density (persons/km^2^) distributions by urban class and by MSA population size classes, for 1990 and 2010 (national block-level comparison in the right-most column; MSA-2000 boundaries used).

When we classify MSAs into groups according to total population sizes, we find remarkable differences. Figure 2 shows that smaller MSAs tend to have significantly greater shares of their population classified as *UPO* (officially urban but sparsely built-up) than do more populous-MSAs; conversely, more-populous MSAs have greater shares of population and land area in the *UAg* category. As MSA population size increases, population density also clearly increases in *UAg* areas, but interestingly remains fairly constant in *UPO* areas.

Population densities in *UAg* areas are lower overall in 2010 than they were in 1990. However, when organizing the data by population size, we see that the most prominent decline occurs in the largest MSAs, those with more than 5 million persons. This may suggest that large cities within large MSAs are becoming somewhat less compact in their cores which would imply differential urban transformations by MSA size. Changes in population density may also result from new, lower-density, urban-designated places being added to MSAs through revised census definitions or from temporal mismatches between GHSL 2014 and the 2010 census data.

If we adopt a more inclusive built-up threshold measure (not shown), we find in MSAs of all sizes that a much larger fraction of the population lives in areas of *UAg*. Furthermore, both *UAg* and *BULO* tend to occupy proportionally more area at lower GHSL threshold values, as would be expected. Alternative thresholds may also affect what inferences can be drawn from changing population density and compactness to characterize processes of infill and expansion. Despite evidence on the scaling properties of population size of cities^29,30^ such heterogeneities in population size and density of cities are highly critical for a detailed understanding of urban structure^31^.

### Size and density-dependent associations exist between urban classes across metropolitan areas

**Fig. 3:**
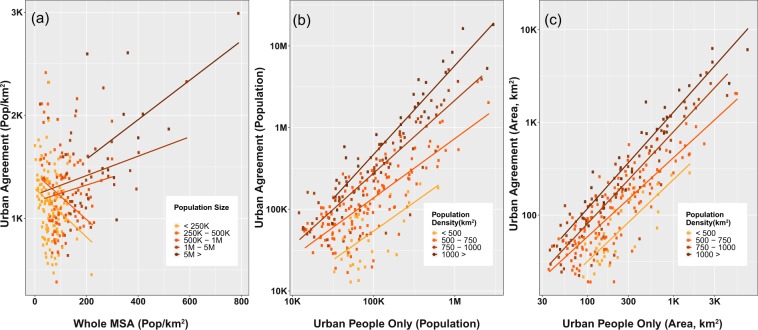
Depictions of relationships between urban classes. Relationships between (**a**) population densities in urban agreement (*UAg*) and whole MSAs, distinguished for different MSA population sizes (indicated by point colors and best fit lines), (**b**) population size in urban agreement (*UAg*) and urban people only (*UPO*); (**c**) area of urban agreement (*UAg*) and urban people only (*UPO*) (for panels b and c, different colors of points indicate MSAs in different population density categories; best fit lines are computed for all MSA points that belong to the same density category; numbers are log-transformed).

We identified inter-class associations across different categories of MSA population size, and population density (Fig. 3) that expose the variety of MSA compositions in the US and shed light on the complexity of metro areas of different characteristics. We found notable levels of sensitivity in the resulting associations along an MSA size gradient. For example, Fig. 3a shows the relationship between all-MSA population density and the density of core-urban (*UAg*) areas. We use all MSA land in this comparison as it is the conventional approach when analysts do not use spatial data to inform their question. However, spatial information is necessary to partition and refine the land area within MSAs to different urban classes and rural land. In larger MSAs, those with more than 500,000 persons, urban population density (within *UAg*) is positively associated with overall population density. But for smaller MSAs, the opposite is true. Overall population density declines as population density in the core urban class rises. When comparing the population density in areas of all urban-classified land (*UI*) to density in all areas within MSAs, similar relationships can be seen for different size categories but the slopes are slightly less steep (whether upward or downward).

As expected, MSAs with larger populations and area in the areas of urban agreement also have larger populations and land area in classes of urban people only and built-up land only. While these associations are similar, intercepts of the best-fit lines vary across groups of population density, shown for population size (Fig. 3b) and class area (Fig. 3c). For example, the most densely populated MSAs (>1,000 persons per square km) have more dwellers in core urban areas relative to dwellers in the urban people only class than those in less densely populated MSAs. These density gradients are not visible for the associations between *UPO* and *BULO* classes.

We also found consistent patterns across MSA size categories in the degree of built-up by urban class for a given GHSL threshold. As MSA population size increases, the distribution of these built-up percentages increases and tightens (higher means, lower variances). The lower ends of built-up distributions within MSAs seem to be less impacted by the omission errors in built-up land classifications in rural settings as described in recent accuracy assessments^32^, likely a consequence of the higher level of built-up density in general that makes up an MSA from the outset.

### Urban class compositions across US metropolitan areas are complex and diverse

**Fig. 4:**
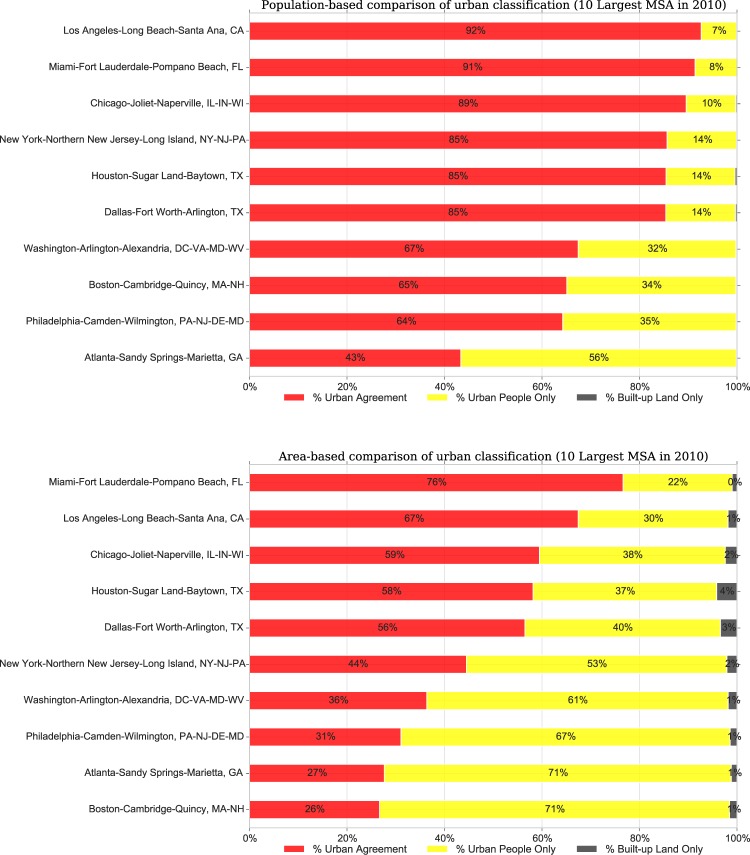
Population and land area by urban classes in 2010 (within 2000 MSA boundaries) for the 10 largest MSAs.

**Fig. 5:**
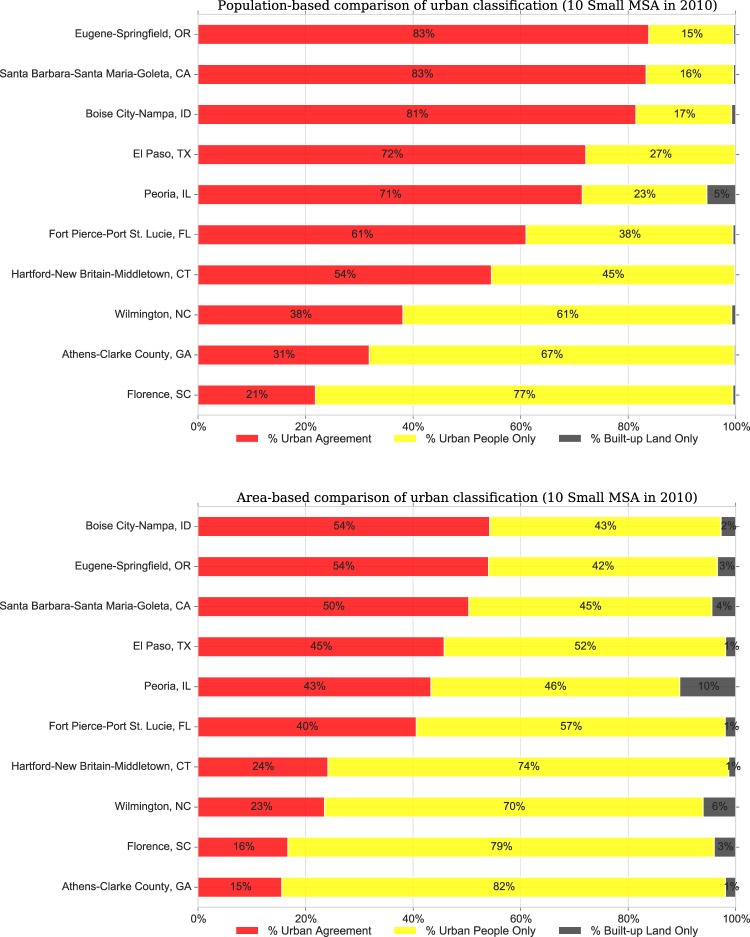
Population and land Area by urban classes in 2010 (within 2000 MSA boundaries) for 10 small MSAs.

**Fig. 6:**
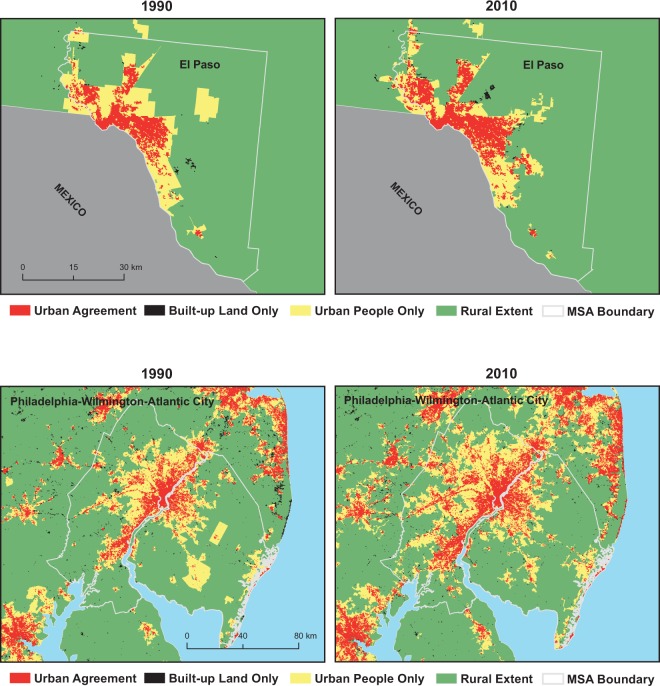
Maps of urban classes in Metropolitan Statistical Areas (MSAs). Urban classifications in 1990 (left) and 2010 (right) using year-2000 MSA boundaries, for one large CMSA (Philadelphia, PA; bottom row) and one small MSA (El Paso, TX; upper row).

We assessed the compositions and distributions of urban classes in all US metro areas in 2010 and found a high degree of heterogeneity of urban patterns across MSAs. To better understand this heterogeneity and the potential implications of urban form on urban change, we focused on 20 selected MSAs, 10 large and 10 small ones that differ widely in size, form and development history. Figure 4 shows, for the 10 largest MSAs, the population (upper panel) and land area (lower panel) in each of the urban classes (*UAg*, *BULO*, *UPO*) in percent in 2010 using fixed-year (i.e., 2000) MSA boundaries. Each of these 10 MSAs, which together represent a population of over 90 million persons (close to 30% of the US population), were paired with one smaller, nearby MSA (Fig. 5) to illustrate the level of variation in the presented urban classification. For example, the Los Angeles-Long Beach, CA MSA was paired with the Santa Barbara-Goleta, CA MSA; Atlanta is paired with Athens, GA. Example maps of urban class distributions are shown in Fig. 6 for El Paso, TX and Philadelphia, PA for 1990 and 2010. The additional 18 example MSAs are shown in Supplementary Figs. S1–S14. The proportions of urban classes in each MSA are shown in Supplementary Figs. S15 and S16.

We find a notable degree of heterogeneity among these MSAs in both population and land area, with more variation evident among the smaller MSAs than the larger ones. Eight of the 10 largest MSAs have about 75% or more of their population living in the *UAg* class. The Atlanta-Sandy Springs-Marietta, GA MSA is the only one with less than half of its population in areas of *UAg* and more people living in areas classified as *UPO*. This conforms to the oft-cited description of Atlanta as the sprawling American city^33^. All large MSAs have very small fractions (around 1% or less) of the population living in *BULO*.

While most of the smaller counterparts to the largest MSAs (Fig. 5) also show an overwhelming majority of their populations in areas of *UAg*, it is to a lesser degree. Not surprisingly, in the counterpart to the Atlanta MSA, the Athens-Clarke County, GA MSA, the vast majority of the population (76%) lives in *UPO* areas. These smaller MSAs have somewhat larger fractions of their population living in areas of *BULO*. The MSAs for Boise (ID), Wilmington (NC), and Florence (SC) all have less than half of their population (and even smaller fractions of their land area) in areas of *UAg*. In contrast to population, the areas of the different classes show greater variation even within the largest MSAs possibly a function of county size in different regions of the US While five of the largest MSAs have the majority of their land area classified as *UAg*, only Miami has more than two-thirds of its land area in *UAg*. The New York City MSA has just less than half of its land area in areas of *UAg*, and slightly more than half in areas of *UPO*.

Notably, the shares of *UPO* – areas that are more suburban in nature – are much more varied even in large MSAs. While the fraction of the land area in these large MSAs that is classified as *BULO* is higher than the fraction of population in *BULO*, these shares are still quite small, reaching 4% only in Houston. In contrast, the smaller MSAs have larger percentages of areas classified as *BULO* than their larger counterparts, but the percentages remain within single digits. Whereas the land area fractions by MSA-size categories showed fairly constant distributions, each individual MSA exhibits its own pattern. These patterns likely have implications for the ways in which MSAs transform from smaller cities into regions, and for the transportation and other amenities within larger commuting zones that serve them^6^.

## Discussion and Conclusions

In this study, the integration of remote sensing-derived, built-up land layers such as GHSL, with demographic data and census classifications of urban locations has enabled us to combine population and land-cover perspectives of urban-ness to reveal the variation in urban structure across Metropolitan Statistical Areas in the United States at different points in time. Our results suggest great potential in pursuing this blended population-land cover approach. The use of remote sensing derived settlement layers enriches the way urban concepts can be shaped, refined and characterized. A recent comparison of the Census Bureau’s urban areas and clusters with the European Commission (EC)’s new Degree of Urbanization data–a blended model of population and GHSL^19^–strongly supports our findings. They report strong agreement in the urban core–which would correspond to our areas of *Urban Agreement*–but find notable differences between the official census definition and those of the EC approach in areas of lower population densities, including suburbs and small towns. The focus on MSAs in the present study provided a unique picture of the diversity of urban patterns found in such larger commuting centers. By exposing differences in urban form within consistent reference extents we were able to better understand their implications for change in urban structure possibly pointing to areas where refinements in measurement would be warranted.

Deriving and evaluating the urban classes of *UAg*, *UPO*, *BULO* and *RE* helped to reveal consistent patterns but also considerable heterogeneity among MSAs and enabled us to characterize urban change over space and time. Areas of urban agreement–i.e., where satellite-based indicators of building density and census-based designations agree–account for somewhat more than three quarters of MSA urban inclusive (*UI*) population and 40% of the *UI* land, in 2010. These areas of agreement tend to have very high fractions of built-up land, regardless of the MSA size class and exhibit population densities three to four times higher than those found in areas classified as urban only by the census. Large MSAs–those with populations greater than 5 million–have population densities in their areas of urban agreement considerably higher than in all smaller MSAs. This difference is most pronounced in 1990, with clear density declines seen by 2010. Like other global metro areas^24,34,35^, urban population growth and land expansion appear to be accompanied by declines in population density in the most built-up parts of these large MSAs.

When looking at inter-class relationships, we found that within groups of MSAs with similar population density, MSA population size and areal extent of the core urban class (*UAg*) is positively related to population size and area, respectively, in other urban classes (e.g., *UPO*). However, in contrast, for population density we identified highly variable inter-class associations across groups of MSAs with similar population size. These variations expose the inherently complex relationships between land area, population size and population density of urban classes and align with a high degree of diversity in urban form and structure across MSAs that can, in part, be seen as a consequence of topographical constraints as well as historical development trajectories of individual MSAs. While *UAg* areas are almost always at the core of the urban extent with the highest population density, and surrounded by areas of *UPO*, the relative size (proportion) and shapes of the different classes varies by MSA. Some MSAs appear to have a more compact profile (for example, with larger fractions of *UAg*) than others, which provides a strong indication of the existence of different types of metro areas. For example, many large MSAs such as Miami, Los Angeles, and New York exhibit very high proportions of both population and land in areas of *urban agreement*, while others, notably Atlanta, Boston and many smaller MSAs show much higher shares of population and land classified as urban people only.

While combining census and satellite data successfully revealed nuanced characterizations of urban form and systematic evaluation of urban development patterns, these complexities expose the inherent difficulties in treating urban as a “unifiable” concept. The presented results draw a unique picture of how different urban environments can be in the United States (and possibly elsewhere) by combining perspectives of land and population. We believe that these insights can contribute to consistent but more differentiated definitions and characterizations of urban systems.

There are several notable limitations of this study that point to future research opportunities. *First*, the assumption of uniformity in population density within a census block is a simplification because population density is likely to be higher within the built-up portions of the block indicated by GHSL. The resulting underestimation of the population in areas of *UAg* and *BULO* and overestimation in areas of *UPO* represents a limitation but is mitigated by the small size of census blocks. In future work, we will apply dasymetric models^36^ to improve population allocation^37,38^. *Second*, our approach does not account for changes in block boundaries but instead analyzes block parts in different points in time, assuming that such block changes are a direct function of urbanization (such as administrative reclassification). Future research will apply areal interpolation methods^39,40^ to create consistent target units for analytical comparison over time. *Third*, for MSAs, we have dealt with boundary change by adopting a fixed geography – using the 2000 MSA boundaries – but this choice imposes some theoretical restrictions on estimates of change. Administrative units such as MSAs are intrinsically land-expansive aggregations that are routinely subject to change. Our need to use a fixed geography here is a caution to those who use MSA area as denominator in land-based calculations (e.g., population densities) because the land-area associated with MSAs may change radically from one census to the next.

Our study provides a strong indication of the potential of using GHSL (or similar products) in conjunction with census data to model the extent and change of urban centers. More complex measures of density, connectivity and proximity derived from settlement layers for different thresholds and over time may be useful to measure different types of urban development by enriching the urban classes described here. Such an effort would open the possibility for comparisons among urban centers worldwide using consistent definitions, which may result in more reliable representations of urban land and population. Such future efforts may have applicability to low- and middle-income countries that are often data-poor^17,41,42^ or as temporally-consistent measures even in data-rich settings.

Related to the above, GHSL may also hold promise for predictions of future urbanization and urban spatial patterns. For example, non-urban but substantially built-up places show a non-trivial likelihood of being classified as census-urban a decade later, suggesting that areas of built-up land only (i.e., areas built-up but officially classified as rural) may represent a leading edge of urban change resulting in *in situ* urban transitions often seen on the urban fringe. We argue that combining these insights with additional ancillary data representing roads or similar measures of connectivity^43^ along with increasing knowledge of the geographical correlates of population distributions in general^44^ and specifically in different regions, will open new opportunities of projecting urban population and urban land classes.

## Data and Methods

Below, we review relevant key concepts in defining urban land and urban people, and discuss the data sources and measures that are used in the analysis. We then describe our method for constructing the spatial classification of urban land and population within MSAs for different census years.

### Context, definitions and data

Recent research has used remote-sensing derived data (e.g., land-cover or night-time lights) to delineate urban boundaries or other spatial features and combine them with social-science data to study urban systems change^17,33,45^ and understand urbanization and social vulnerability with respect to climate change and related risks^46–50^. However, the direct comparisons of satellite with census data^22^ remain uncommon. Below, we reflect on how disparate communities conceptualize urban areas and describe our three distinct data inputs, representing constructs of urban population, metropolitan commuting centers and a proxy for urban land (see Table 1).

#### Different perspectives and measurement of urban areas

Depictions of urban land are commonly derived from satellite images, with urban areas typically defined by land-use and land-cover measures^15,38,50–52^. In contrast, the social science research community conceptualizes urban areas in demographic and social terms: urban population is mainly defined as a function of governmental jurisdictions and boundaries, population density, spatial contiguity and size, sometimes supplemented with socioeconomic indicators of connectivity such as commuting zones^4,53–57^. According to the Congressional Research Service, the first official US definitions of standard metropolitan areas emerged in the late 1940s^58^, when the US Bureau of the Budget (which in 1970 became the Office of Management and Budget (OMB)) sought to establish a measure of order and consistency to the various criteria by which government funds were allocated to “metropolitan” areas or to the rural communities linked to them. The definitional specifics have evolved in sophistication over the ensuing decades, but certain principles remain largely intact: the US Census Bureau measures of urban land and people have increasingly focused on population density, contiguity and size, irrespective of formal jurisdictional boundaries. With the advent of census data on commuting in the US, it became possible to consider the inclusion of counties that lack urban centers if a sufficient percentage of their residents partake in urban life by way of commuting. In short, the outer boundaries of MSAs are comprised of jurisdictional boundaries (i.e., counties), but their internal composition of urban and rural land and people is defined in terms of the Census Bureau criteria.

Both social and physical science analytic perspectives have emphasized the disconnect between complex urban concepts and the limited abilities of the available data to measure these concepts^59,60^. Due to the variation of urban concepts between academic disciplines and different national urban classification systems^1^, there are no universal conventions for measuring urban people or urban land, consistently, across countries and time. As such, comparison of urbanization trends across space and time can produce misleading results. Thus, many researchers have noted an urgent need to invest in the development of a more consistent approach to measuring urban concepts^5,29^.

#### Census block-level data

The census block is the finest-grained spatial unit used by the United States Census with complete geographic coverage over the last three decennial censuses (1990, 2000, and 2010). Census blocks are delineated by both man-made and physical characteristics of the landscape, such as roads and rivers as well as legal and administrative boundaries; in densely populated urban areas they typically comprise actual city blocks. They can vary widely in area and total population, ranging from zero to several hundred people. In response to changes in development and population density, the number of census blocks has increased, especially in, and in close proximity to, urban centers.

At each census, blocks and their population are defined as urban or rural according to criteria that have changed over time (see Table 2)^3^. In the 1990 census, all blocks within an area that met the criteria of an agglomeration (urbanized areas, or UAs) i.e., census incorporated or designated places of more than 50,000 persons, were defined as urban. Any census blocks adjacent to such qualifying territory with a population density over 1,000 people/mi² were also included in the larger urbanized area. Outside of urbanized areas, blocks were defined as urban if they were part of incorporated places or census-designated places with a population greater than 2,500^61^. The use of census incorporated/designated places (cities) as a starting point for constructing UAs was dropped in the 2000 census and blocks were the primary building units. A UA was defined as a contiguous set of blocks with a population density >1,000 people/mi² and a total population of >50,000. The count-oriented definition for urban places was also dropped in favor of identifying urban clusters (UCs), defined as a core set of contiguous census blocks with a density greater than 1,000 people/mi² and a total population of 2,500–49,999. Any blocks within UAs and UCs and those within close proximity (within 2.5 miles) to UAs and UCs were defined as urban if their population density exceeded 500 people/mi². For 2010, the 2000 urban classification scheme was further amended to include some categories of land in industrial and commercial use: non-residential blocks mainly covered by impervious surfaces (pavement, parking lots, and airports) in close proximity (within 0.25 miles) to populated urban blocks within UAs and UCs. The Census Bureau drew its impervious surface measures from Landsat imagery prepared as a by-product of the National Land Cover Database^3^.

**Table 2:** Input data by type, source, and temporal and spatial resolution.

Feature	Spatial Product	Years	Urban Proxy Definition based on…	Resolution
Census geography-Urban People	US Census blocks	1990	Population density and count, proximity: jumps up to 1.5 miles	Variable (delineated by man-made and physical features of the landscape)
		2000	na	
		2010	Population count, density, land-use; proximity: hops up to 2.5 miles	
OMB geography-Commuting Area	Metropolitan Statistical Areas	1990	Population count, population density, employment connectivity (commuting)	Variable (mainly delineated by county and aggregate county boundaries)
		na		
		2010		
Satellite-based urban proxies-Urban Land	Global Human Settlement Layer	c. 1975, 1990, 2000, 2014	Measure of built-up land cover. Urban extents are constructed based on grid cell values meeting a 25%, 40%, or 50% built-up threshold.	original: 30 meters original GHSL, aggregated: 250 meters

#### Metropolitan statistical areas (MSAs)

Since 2003, the US Office of Management and Budget (OMB) defines metropolitan statistical areas as collections of governmental jurisdictions, as a county or sets of contiguous counties that contain a UA of more than 50,000 persons. MSAs may include outlying counties if more than one-quarter of residents are in- or out-commuters.

MSA boundaries change over time, both as a consequence of structural changes in the population and changes in the conceptualization and measurement of urbanization and commuting areas^62^. In 2010, there were 363 MSAs in the continental United States, up from 274 in 2000 in part due to splitting larger MSAs into smaller ones (Supplementary Fig. S17). MSA boundaries for our three study years are shown in Supplementary Fig. S17 to highlight the need for addressing such boundary changes and their complications^5^. While the large majority of the US population lives in MSAs, urban areas within these units comprise only 2.6% of the country’s total area in 2000.

Metropolitan areas may be viewed as if they were broad planning units for urban centers and their connected populations, with an explicit allowance made for the rural-dwellers who are linked to such urban centers^8,63–66^. Studies of the US urban population frequently adopt metropolitan areas as their analytical units in large part because the data they rely on use MSAs to proxy for urban locations^5,6,64,67–70^.

While MSAs make for an appealing analytical unit, their spatial extent and internal heterogeneity can be considerable. For example, 12.6% of the MSA population in 2000 is designated as rural (Table 1), which equates to roughly 50% of the total rural US population. Furthermore, rural-designated land occupies far more of the metropolitan area landscape than does urban-designated land. Finally, of note, is that 16% of census-designated urban blocks and 10 percent of the urban population in 2000 were located outside metropolitan areas^71^ (Table 3).

**Table 3:** Comparison of Urban Population (census-defined) within and beyond MSAs between period-specific and fixed 2000 MSA Boundaries for the contiguous US Percentages represent fraction of the total population or land area respectively.

	MSA Boundaries		1990		2000		2010	
			Count	%	Count	%	Count	%
Population (000 s)	Year-specific	Within MSA	164,953	66.9%	198,352	71.0%	242,561	79.1%
		Outside MSA	20,439	8.3%	22,399	8.0%	4,956	1.6%
	Year 2000	Within MSA	167,750	68.0%	198,352	71.0%	223,097	72.8%
		Outside MSA	17,642	7.2%	22,399	8.0%	24,420	8.0%
Area (km^2^)	Year-specific	Within MSA	180,413	2.3%	201,213	2.6%	270,078	3.5%
		Outside MSA	44,734	0.6%	38,998	0.5%	9,188	0.1%
	Year 2000	Within MSA	187,182	2.4%	201,213	2.6%	235,326	3.0%
		Outside MSA	37,964	0.5%	38,998	0.5%	43,940	0.6%

#### The global human settlement layer

The Global Human Settlement Layer (GHSL), produced by the Joint Research Center (JRC) of the European Commission, consists of built-up land layers, encompassing a time period of 40 years (1975, 1990, 2000, and 2014) at fine spatial resolution (approximately 30 meters, aggregated to 250 meters). The original data are binary, representing the presence or absence of a built structure in a 30 meter grid cell^20,21,72–74^; a cell is coded as built-up if it overlaps with a built structure or impervious surface (but not roads). The aggregated cells (250 m) are assigned the proportion of built-up land as raster value. We use the 250 m grids (rather than the native 30 m grids) because these translate into proportions built-up and because most census blocks are larger than 250 m (except in dense cities) and thus represent compatible scales. Recent validation efforts have reported acceptable levels of accuracy of the GHSL except in rural regions^32,75^.

Other broadly similar global data products suffer from limitations either in terms of coarse spatial resolution such as the Global Rural Urban Mapping Project, GRUMP^45,47^, or limited temporal coverage such as GlobCover^76^, the Global Urban Footprint^77^ or GHSL Sentinel^74^. The GHSL, therefore, offers new opportunities for usages where the analysis of temporal change is required such as in population forecasting, and in the analysis and modelling of urban dynamics and land use change.

## Approach

For each MSA, we overlaid spatial census block data that carry official designations of urban and rural with GHSL-derived binary proxies of built-up/non-built-up area, to produce an urban classification, following a conceptual model shown in Fig. 1. For each of the resulting classes, we estimated the area and population for 1990, 2000 and 2010 in order to examine differences in the resulting patterns over time and across all MSAs. In order to keep the analytical extent consistent over time, we fixed the geometry to the 2000 MSA extents. This way we mitigated the effect of changing MSA boundaries and were able to use comparable estimates of urban people and urban land for different points in time.

### Urban classes in MSAs

We restricted our analysis to the MSAs in the continental United States, and overlaid the built-up land layers derived from GHSL raster data (250 meter grid cells) on the census blocks, which are wholly designated as urban or rural. For the built-up land layer, we applied the 50% threshold of built-up proportions to each raster cell to classify it as built-up or not built-up (binary codes) for each point in time. The 50% threshold is the most commonly-cited threshold for defining urban land from GHSL^17^ but other, more inclusive thresholds (e.g., 25% and 1%) can be used to test for sensitivity.

The following geoprocessing steps, which were applied for each point in time (details can be found elsewhere^22^), created several classes of land within each MSA by overlaying the binary built-up land measure with a binary census-block classification: First, we identified all urban census blocks to determine people-based urban extents (Fig. 1a). Second, we used a threshold of 50% proportions built-up to create land-based estimates of “highly built-up” extents (Fig. 1b). Third, we ran the union of the people-based urban extents and land-based built-up extents to generate a third layer, *urban inclusive (UI)*, any land area that is part of either of the two binary layers (Fig. 1c). Fourth, we defined the *“rural extents” (RE)* layer (green in Fig. 1) as all land that is not part of either of the binary layers.

The decomposition of the *UI* areas into classes that reflect people- and land-based conceptualizations resulted in three additional layers. A class of *urban agreement (UAg)* is the intersection of the people-based urban extents (census blocks) and land-based (GHSL) built-up extents (Fig. 1d). We then erased the area of the *urban agreement* class from the people-based urban extent to create a class which meets only the census (people)-based definition of urban, entitled *urban people only (UPO)* (Fig. 1e). Finally, we erased the areas of urban agreement from the GHSL-based built-up extent, creating the *built-up land only (BULO)* layer (Fig. 1f). The classes *UAg*, *UPO*, *BULO*, and *RE* were used to compose a four-class distribution within all MSAs (Fig. 1g).

### Estimating area of and people in urban classes and rural extents of MSAs 1990–2010

Using these classifications, we measured areas of all classes in all points in time, 1990, 2000 and 2010 within MSA boundaries of the Census 2000. At each point in time, we allocated block-level population counts to all urban/rural classes within blocks based on area proportions of these classes, which required an assumption of uniform population distribution within each block^78,79^. This areal weighting approach assumes constant population densities within blocks and thus may underestimate population in highly built-up sections of blocks and overestimate population in less developed sections. However, since we are considering MSAs, and thus generally higher urbanized areas, we expected that such effects would not impact the average estimates of population densities significantly. We conducted all processing steps using Python geoprocessing capabilities of ArcGIS 10.6.1, and the procedure was repeated for each census period.

### Assessing urban compositions within consistent MSA geographies

As described above, the definition of a metro area, and subsequently the number and distribution of metro counties, has varied significantly over the past several decades.

Such inconsistency in metro boundaries is known to impact metrics compiled over time using MSAs as the analytical unit, leading many researchers to “fix” boundaries at one point in time or consider alternative classification schemes^5,48^. This problem also affects this study as temporal analysis cannot be done within the original, changing units but requires the use of consistent boundaries to identify changes that are not due to the redrawing of boundaries alone. We take as a benchmark the MSA geography of the mid-point year (2000). This ‘fixed’ geography allows us to assess the changes in urban population and land area within temporally consistent extents without the need for complex areal interpolations and population allocation models^39,40,80^. Table 3 demonstrates the level of average estimation bias (+2.5% in 1990; −2.7% in 2010) that would be caused on average by using changing MSA boundaries. The area comprised by original MSAs covered just over 1.5 million km² (19% of the country) in 1990, 1.85 million km² in 2000 and 2.3 million km² (29% of the country) in 2010. Population density estimates within original MSAs would thus decline, on average, over the three periods, a function of expanding geographic boundaries, but could fluctuate considerably for individual MSAs. Using consistent boundaries ensures that patterns, counts, and densities remain comparable over time at the expense of missing some parts or including areal proportions that might not be included in the official boundaries before or after. However, the ability to observe and measure how an area is changing within a constant perimeter is crucial and thus an important aspect here.

Using the 2000 MSA boundaries, we created the urban classification (using built-up percentage thresholds of 50%) and applied the areal weighting procedure to estimate population and land area as described above for the full set of MSAs in 1990, 2000 and 2010. This procedure could be repeated using target boundaries of MSAs from any of these years, accordingly, allowing for cross-sectional comparisons at each time period across different regions and metro area types as well as longitudinal comparisons to assess the evolution of urban structure.

## Supplementary information

Supplementary information.

## Data Availability

We used three types of publicly available data in this analysis. First, we employed Census blocks, the finest-grained spatial unit published by the United States decennial census with complete geographic coverage over the last three decennial censuses (1990, 2000, and 2010). The census block boundaries (TIGER/LINE Shapefile format) are available from https://www.census.gov/cgi-bin/geo/shapefiles/index.php. The block-level attributes (population, urban-rural designation) are available from https://factfinder.census.gov/. The National Historical GIS (NHGIS) also makes available census boundary data at https://www.nhgis.org/documentation/gis-data and attributes from https://data2.nhgis.org/main. Second, we used Metropolitan Statistical Area (MSA) boundary data of the year 2000. MSA boundaries change between 1990 and 2010 and we selected the mid-year MSA boundaries (2000) for carrying out the temporal analysis. These boundary files are available in our compressed data directory (see link below) and also from NHGIS: https://www.nhgis.org/user-resources/data-availability. Third, we used the Global Human Settlement Layer (GHSL) at 250 m resolution for the years 1990, 2000, and 2014, available for download from https://ghsl.jrc.ec.europa.eu/ghs_bu.php. The created data layers representing urban classes and rural land 1990–2010 within MSAs are available for download from the figshare public data repository^81^. The data can be downloaded as a 7z archive. Once extracted, the data layers can be found in the *Data* subdirectory which contains a map document (CensusGHSL_MSA.mxd) to open the data and map layouts in an ArcMap project and two subfolders. The *MSA_Boundaries* subfolder contains the MSA boundaries of the year 2000. The *Urban_Classes* subfolder contains the produced urban classes based on block-level census data and GHSL for the years 1990, 2000 and 2010, and for different GHSL built-up thresholds (1%, 25% and 50%). In the *Documentation* subdirectory the user will find a data dictionary, and an illustrative image of the data collection (.png).

## Code availability

The *Code* subdirectory at the above data repository contains the python scripts used to produce the urban class data layers and the zonal statistics tools upon which our analysis is drawn.
