# Supplementary material for: The heterogeneity and change in the urban structure of metropolitan areas in the United States, 1990–2010
Source: Sci Data. 2019 Dec 16;6:321. doi: 10.1038/s41597-019-0329-6 (PMC6915769; doi:10.1038/s41597-019-0329-6)
Supplement: Supplementary file 1 — Supplementary information. [file 41597_2019_329_MOESM1_ESM.pdf]

SUPPLEMENTARY INFORMATION

The heterogeneity and change in the urban structure of metropolitan areas in the United States, 1990-2010

Stefan Leyk, Deborah Balk, Bryan Jones, Mark R. Montgomery and Hasim Engin

TABLE OF CONTENTS:

Figure S1. Urban classification in1990 and 2010, 50% GHSL threshold, with year-2000 MSA boundary; Atlanta and Athens, GA MSAs.

Figure S2. Urban classification in1990 and 2010, 50% GHSL threshold, with year-2000 MSA boundary; Boise, ID MSA.

Figure S3. Urban classification in1990 and 2010, 50% GHSL threshold, with year-2000 MSA boundary; Boston, MA and Hartford, CT MSAs.

Figure S4. Urban classification in1990 and 2010, 50% GHSL threshold, with year-2000 MSA boundary; Chicago, IL MSA.

Figure S5. Urban classification in1990 and 2010, 50% GHSL threshold, with year-2000 MSA boundary; Dallas/Fort Worth, TX MSA.

Figure S6. Urban classification in1990 and 2010, 50% GHSL threshold, with year-2000 MSA boundary; Eugene, OR MSA.

Figure S7. Urban classification in1990 and 2010, 50% GHSL threshold, with year-2000 MSA boundary; Florence, SC MSA.

Figure S8. Urban classification in1990 and 2010, 50% GHSL threshold, with year-2000 MSA boundary; Fort Pierce and Miami/Fort Lauderdale, FL MSAs.

Figure S9. Urban classification in1990 and 2010, 50% GHSL threshold, with year-2000 MSA boundary; Houston, TX MSA.

Figure S10. Urban classification in1990 and 2010, 50% GHSL threshold, with year-2000 MSA boundary; New York, NY MSA

Figure S11. Urban classification in1990 and 2010, 50% GHSL threshold, with year-2000 MSA boundary; Peoria, IL MSA.

Figure S12. Urban classification in 1990 and 2010, 50% GHSL threshold, with year-2000 MSA boundary; Los Angeles and Santa Barbara, MSAs.

Figure S13. Urban classification in1990 and 2010, 50% GHSL threshold, with year-2000 MSA boundary; Washington, DC MSA.

Figure S14. Urban classification in1990 and 2010, 50% GHSL threshold, with year-2000 MSA boundary; Wilmington, NC MSA.

Figure S15. Share of population by urban classification (year-2000 boundaries), 50% GHSL threshold, all MSAs, with total MSA population, and urban population density.

Figure S16. Share of land area by urban classification (year-2000 boundaries), 50% GHSL threshold, all MSAs, with total MSA population, and urban population density.

Figure S17. Change in Metropolitan Statistical Area (MSA) boundaries over Decennial Census periods, contiguous U.S., 1990-2010.

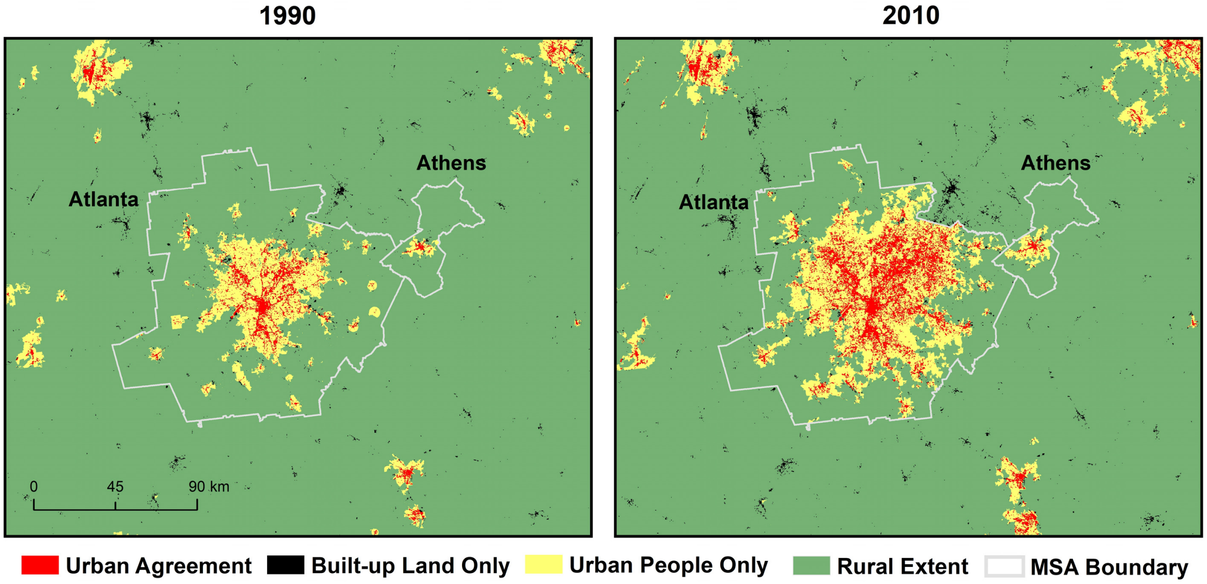

Figure S1. Urban classification in1990 and 2010, 50% GHSL threshold, with year-2000 MSA boundary; Atlanta and Athens, GA MSAs.

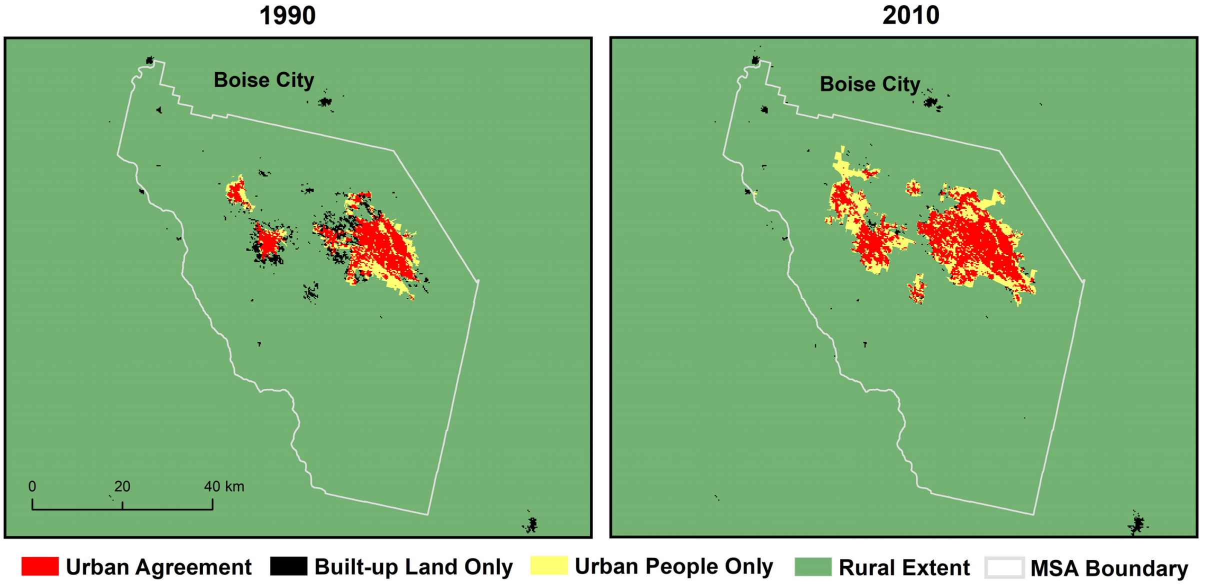

Figure S2. Urban classification in1990 and 2010, 50% GHSL threshold, with year-2000 MSA boundary; Boise, ID MSA.

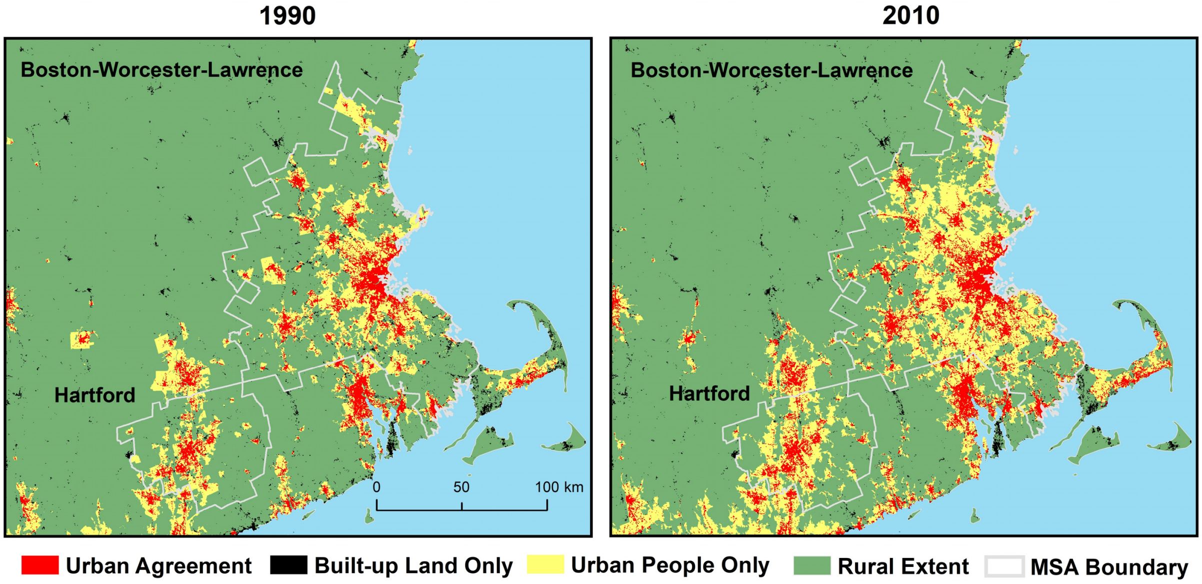

Figure S3. Urban classification in1990 and 2010, 50% GHSL threshold, with year-2000 MSA boundary; Boston, MA and Hartford, CT MSAs.

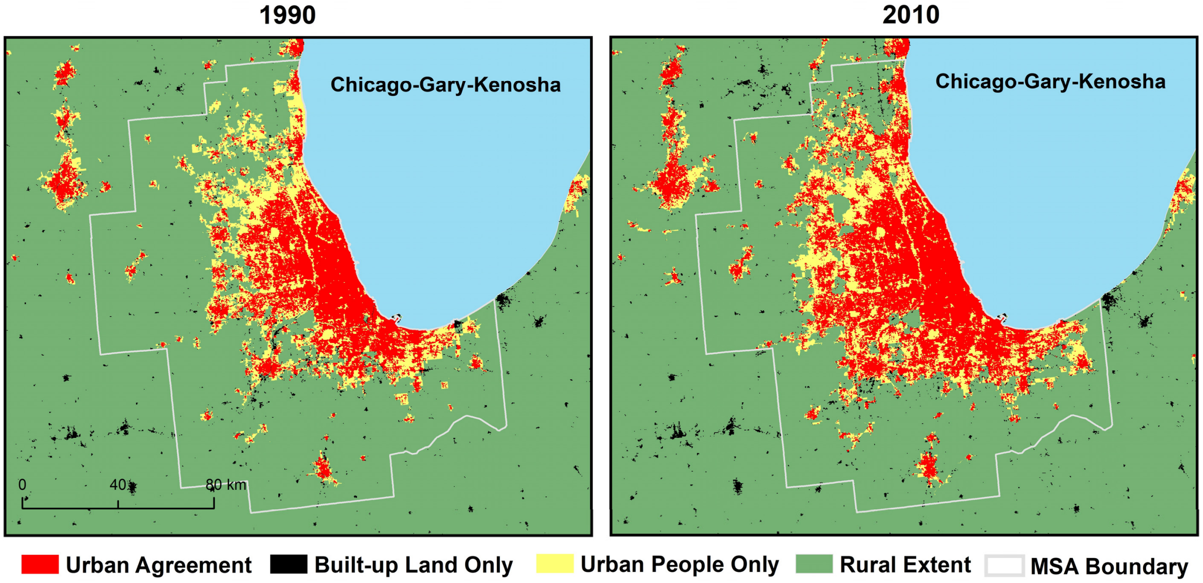

Figure S4. Urban classification in1990 and 2010, 50% GHSL threshold, with year-2000 MSA boundary; Chicago, IL MSA.

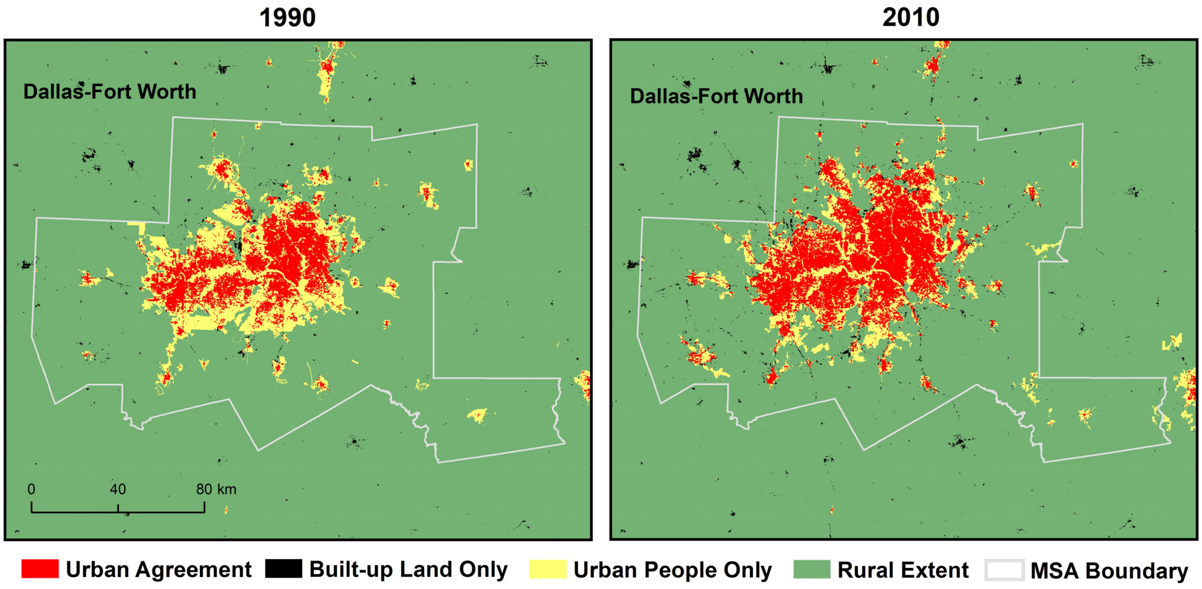

Figure S5. Urban classification in1990 and 2010, 50% GHSL threshold, with year-2000 MSA boundary; Dallas/Fort Worth, TX MSA.

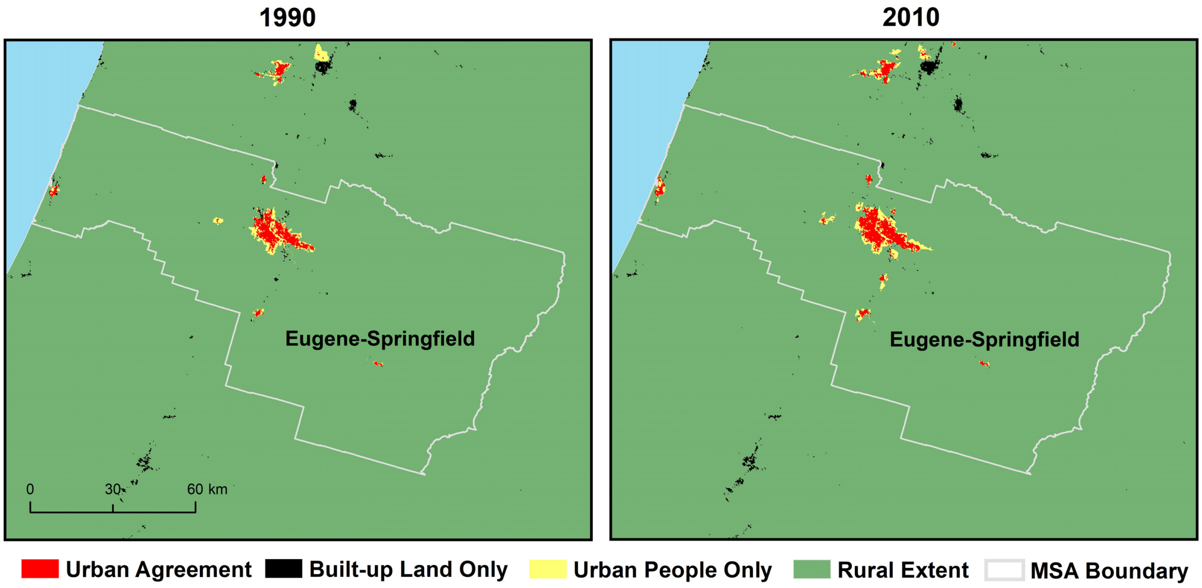

Figure S6. Urban classification in1990 and 2010, 50% GHSL threshold, with year-2000 MSA boundary; Eugene, OR MSA.

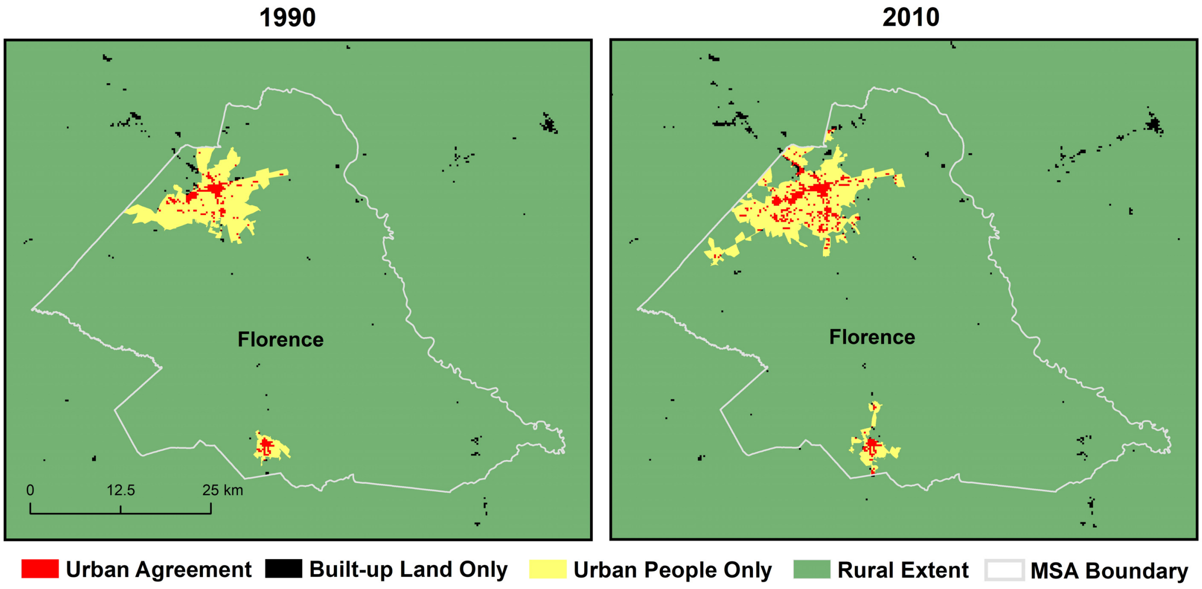

Figure S7. Urban classification in1990 and 2010, 50% GHSL threshold, with year-2000 MSA boundary; Florence, SC MSA.

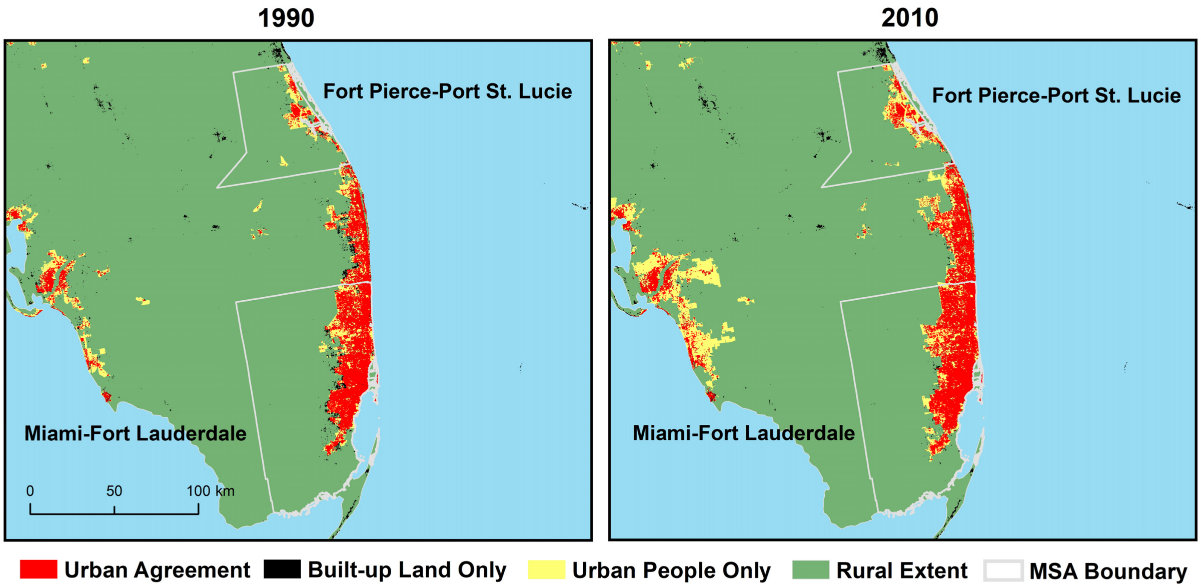

Figure S8. Urban classification in1990 and 2010, 50% GHSL threshold, with year-2000 MSA boundary; Fort Pierce and Miami/Fort Lauderdale, FL MSAs.

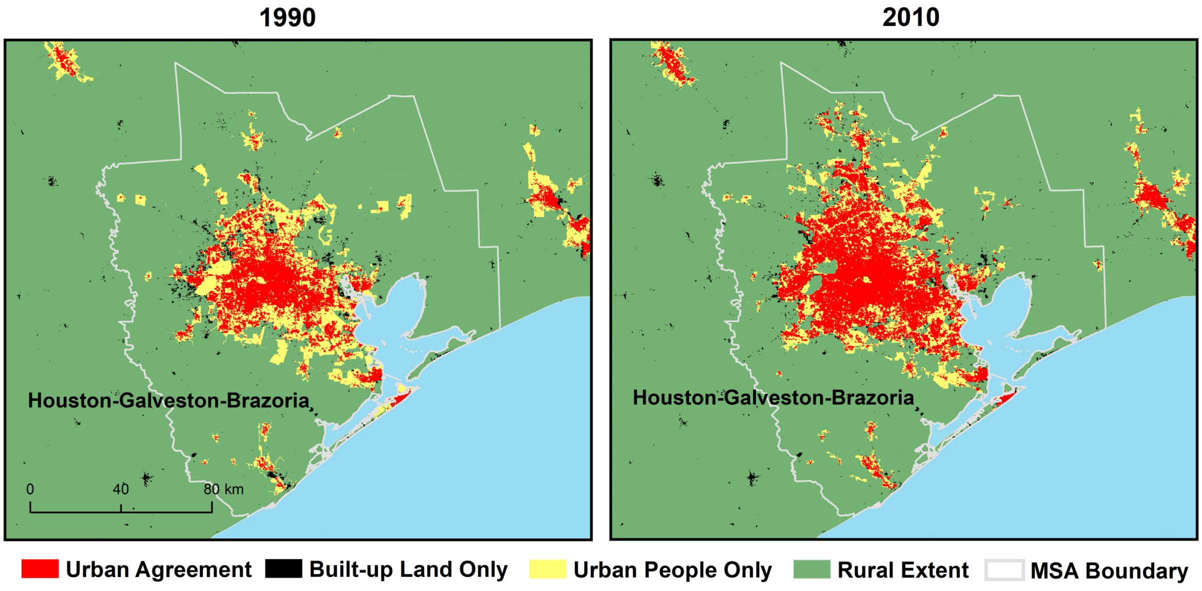

Figure S9. Urban classification in1990 and 2010, 50% GHSL threshold, with year-2000 MSA boundary; Houston, TX MSA.

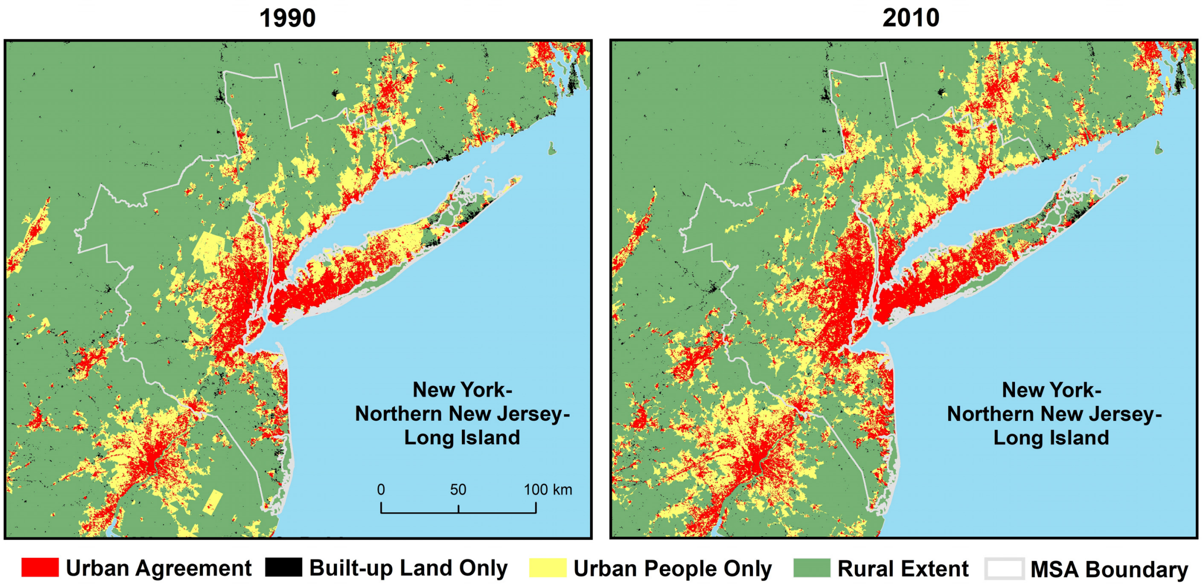

Figure S10. Urban classification in1990 and 2010, 50% GHSL threshold, with year-2000 MSA boundary; New York, NY MSA

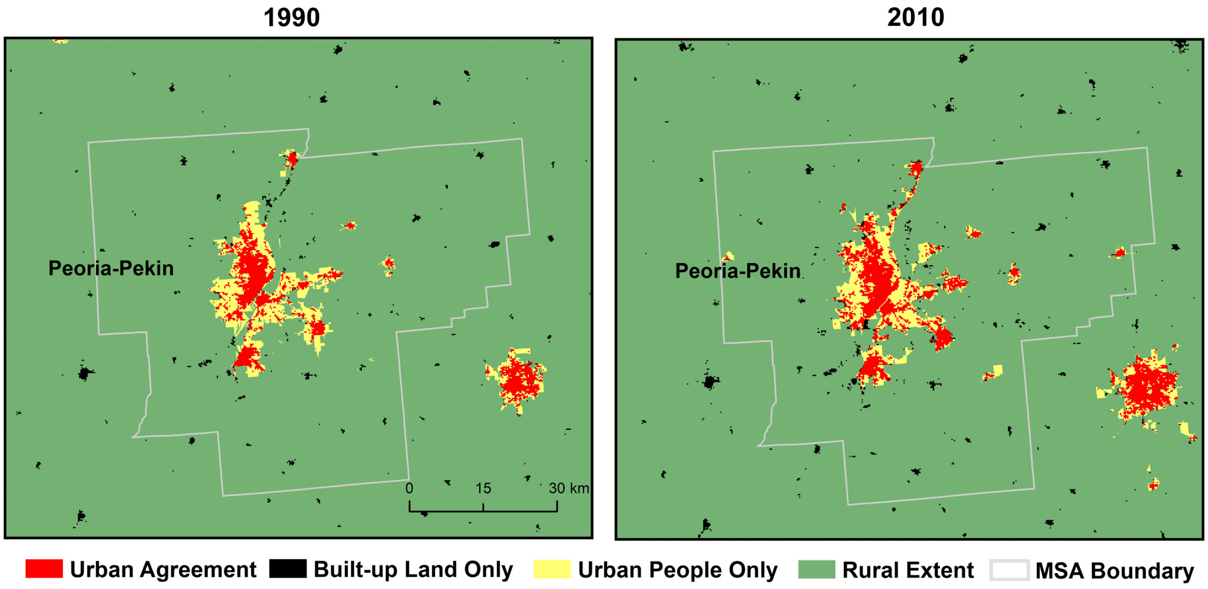

Figure S11. Urban classification in1990 and 2010, 50% GHSL threshold, with year-2000 MSA boundary; Peoria, IL MSA.

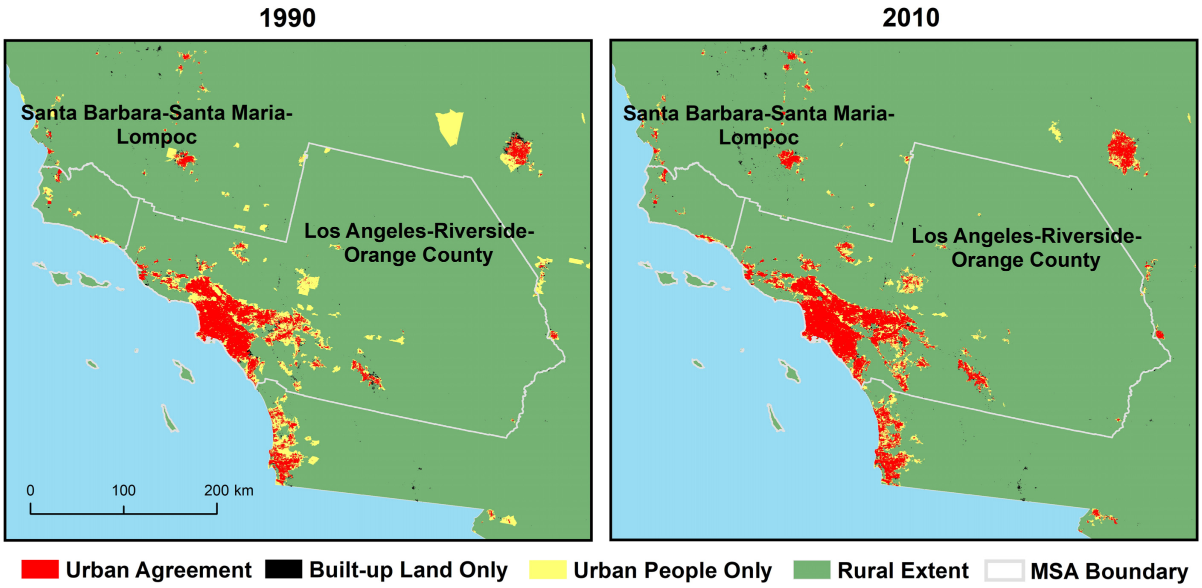

Figure S12. Urban classification in, 1990 and 2010 year-2000 geography, 50% GHSL threshold, with year-2000 MSA boundary; Los Angeles and Santa Barbara, MSAs.

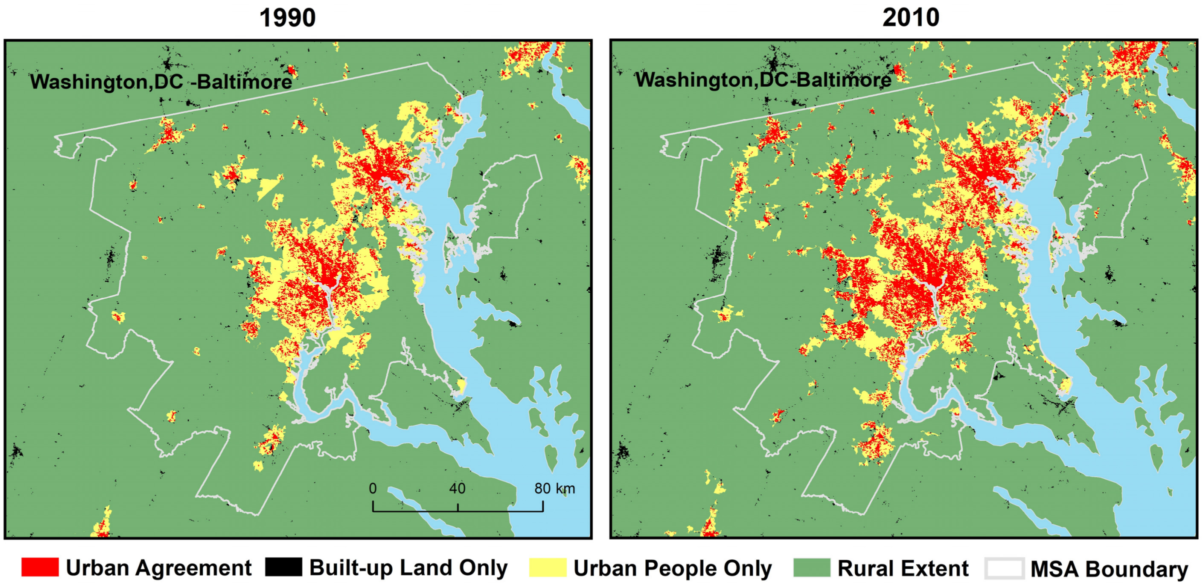

Figure S13. Urban classification in1990 and 2010, 50% GHSL threshold, with year-2000 MSA boundary; Washington, DC MSA.

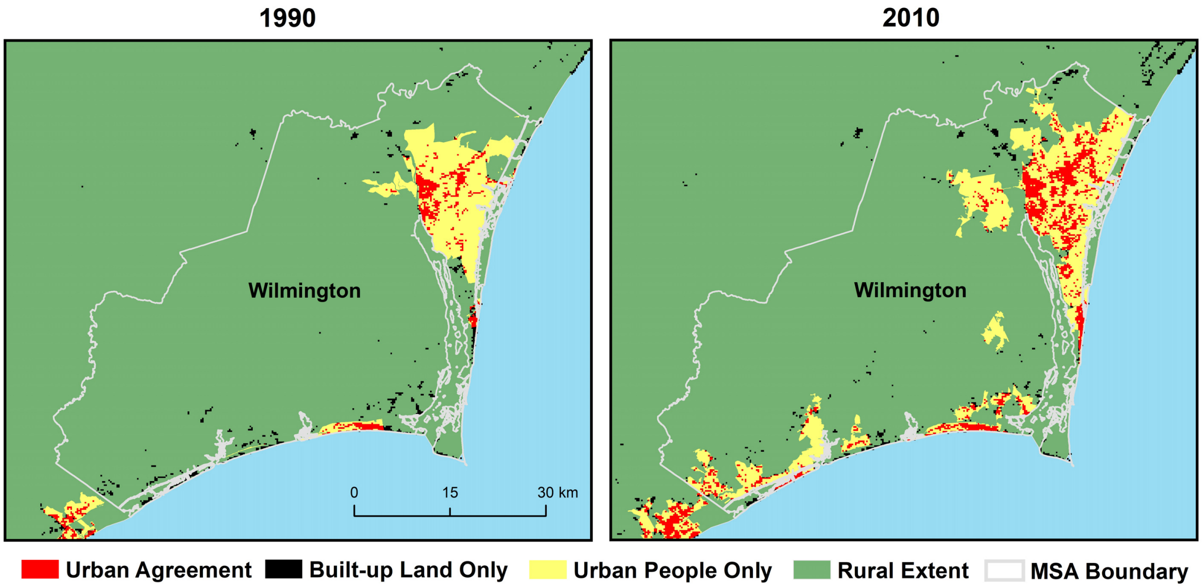

Figure S14. Urban classification in1990 and 2010, 50% GHSL threshold, with year-2000 MSA boundary; Wilmington, NC MSA.

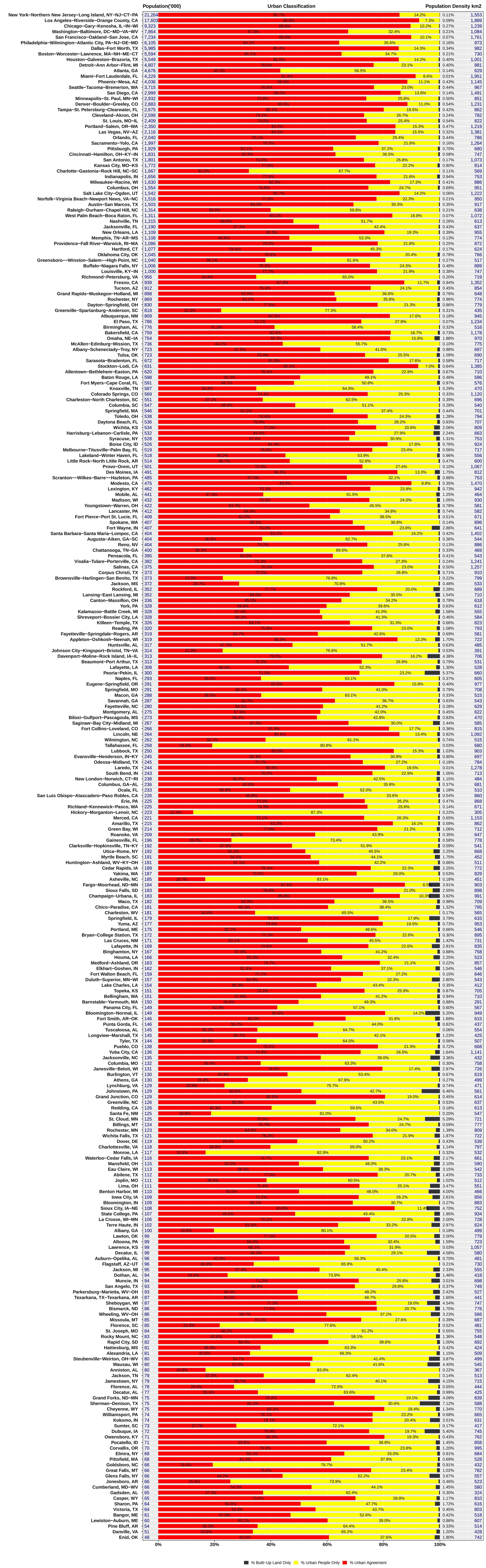

*MSAs are ranked based on population, 2010.
*Rural population is excluded.

Figure S15. Share of population by urban classification (year-2000 boundaries), 50% GHSL threshold, all MSAs, with total MSA population, and urban population density.

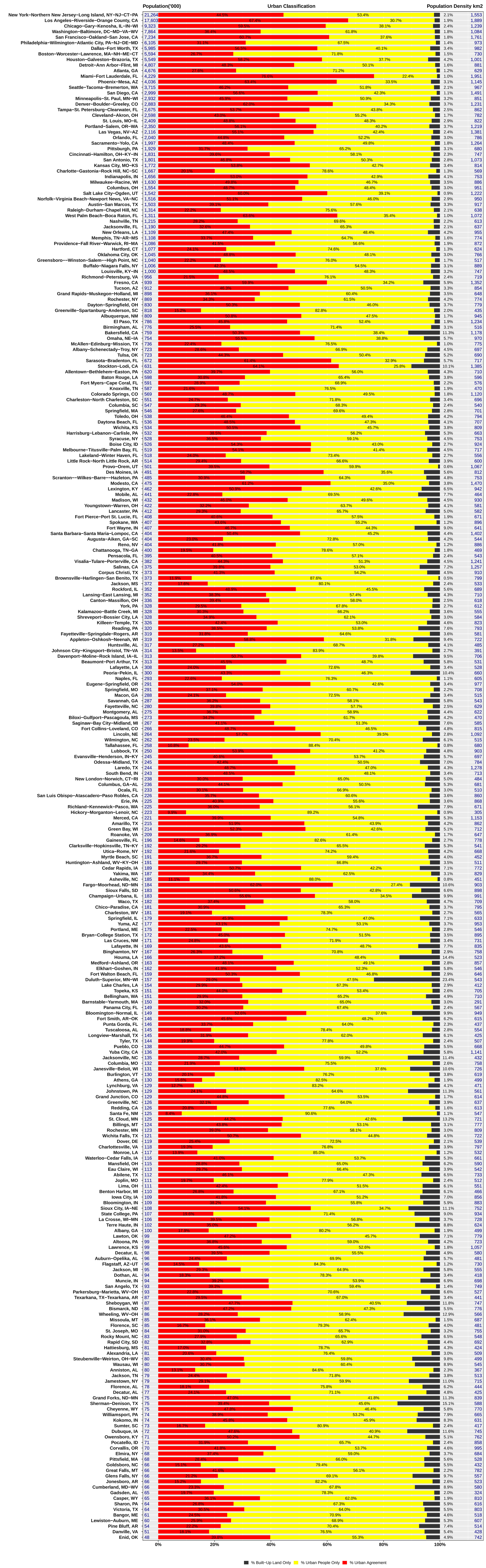

*MSAs are ranked based on population, 2010.
*Rural area is excluded.

Figure S16. Share of land area by urban classification (year-2000 boundaries), 50% GHSL threshold, all MSAs, with total MSA population, and urban population density.

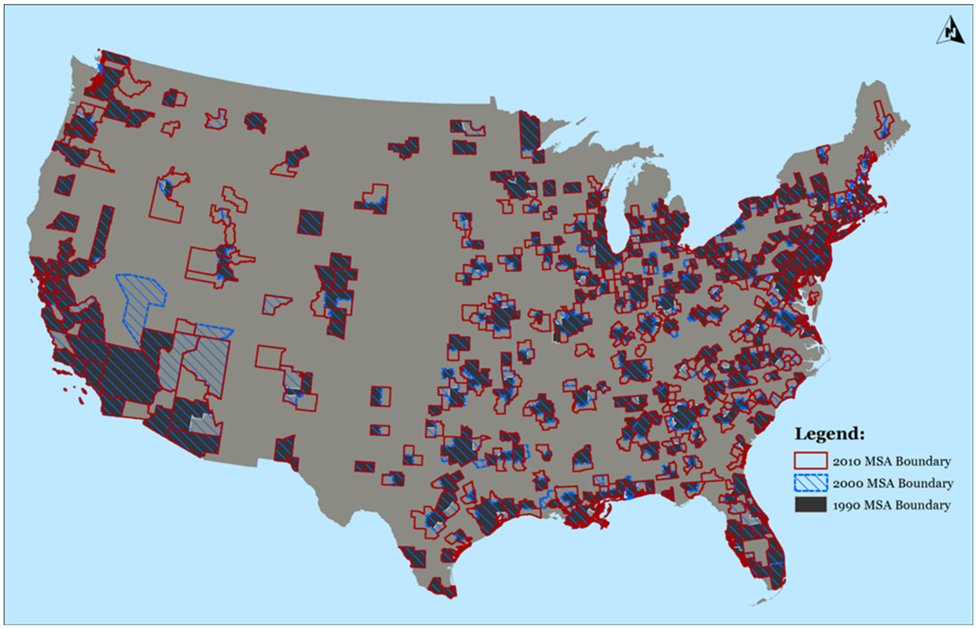

Figure S17. Change in Metropolitan Statistical Area (MSA) boundaries over Decennial Census periods, contiguous U.S., 1990-2010.
